# Assessing landscape N removal in coastal New England catchments using the N-Sink approach with the R Package, *nsink*

**DOI:** 10.12688/f1000research.144100.1

**Published:** 2024-06-07

**Authors:** Dorothy Q Kellogg, Jeffrey W. Hollister, Chester L. Arnold, Arthur J. Gold, Emily H. Wilson, Cary B. Chadwick, David W. Dickson, Qian Lei-Parent, Kenneth J. Forshay

**Affiliations:** 1 Department of Natural Resources Science, University of Rhode Island, Kingston, Rhode Island, 02881, USA; 2 Office of Research and Development, Atlantic Coastal Environmental Sciences Division, US Environmental Protection Agency, Narragansett, RI, 02882, USA; 3 Center for Land Use Education and Research, University of Connecticut, Haddam, Connecticut, 06438, USA; 4 Office of Research and Development, Groundwater Characterization and Remediation Division, US Environmental Protection Agency, Ada, OK, 74820, USA

**Keywords:** Nitrogen, R, watershed, catchment, GIS, landscape N sink, water resources, nsink

## Abstract

**Background:**

Excess nitrogen (N) loading to coastal ecosystems impairs estuarine water quality. Land management decisions made within estuarine watersheds have a direct impact on downstream N delivery. Natural features within watersheds can act as landscape sinks for N, such as wetlands, streams and ponds that transform dissolved N into gaseous N, effectively removing it from the aquatic system. Identifying and evaluating these landscape sinks and their spatial relationship to N sources can help managers understand the effects of alternative decisions on downstream resources.

**Methods:**

The N-Sink approach uses widely available GIS data to identify landscape sinks within HUC-12 (or larger) catchments, estimate their N removal potential and map the effect of those sinks on N movement through the catchment. Static maps are produced to visualize N removal efficiency, transport and delivery, the latter in the form of an index. The R package *nsink* was developed to facilitate data acquisition, processing and visualization.

**Results:**

The R package creates static maps for a specific HUC-12, or users can visit the University of Connecticut website to explore previously mapped areas. Users can also investigate specific flowpaths interactively by clicking on any location within the catchment. A flowpath is generated with a table describing N removal along each segment. We describe the motivation behind developing *nsink*, discuss implementation in R, and present two use case examples. *nsink* is available from https://github.com/USEPA/nsink.

**Conclusions:**

N-Sink is a decision support tool created for local decision-makers to facilitate better understanding of the relationship between land use and downstream N delivery. Local decision-makers that have prioritized N mitigation in their long-term planning can use *nsink* to better understand the potential impact of proposed development projects and zoning variances. Similarly, land trusts and other NGOs interested in N mitigation can use *nsink* to identify high priority areas for acquisition or restoration.

## Introduction

Excess nitrogen (N) delivery via surface water to coastal estuaries contributes to impaired water quality evidenced by excess algal blooms and hypoxia ( Ryther and Dunstan 1971, Nixon 1995, Howarth and Marino 2006). Hydrologic connections and flowpaths are influential in the delivery of nutrients to estuaries ( Mengistu et al. 2020). Identifying landscape N sinks along hydrologic flowpaths – areas where dissolved N is transformed to gaseous N and effectively removed from the aquatic system – and their effect on N delivery at the watershed scale could provide watershed managers, land use planners and conservation organizations additional strategies that target N reductions. N-Sink, and its associated R package *nsink,* were developed to be a screening tool that describes the areas or points within a watershed linked with flow paths where downgradient N sinks process and remove excess N, versus areas where downgradient N sinks are less prevalent or less effective at removing N ( Hollister et al. 2022, R Core Team 2022). This watershed flow path approach provides a means to easily visualize the relative sensitivity of an area within a watershed that may require more aggressive N management at the source, protection of existing N retentive areas, or restoration and construction of downgradient N retentive areas that are needed to reduce N delivery to estuaries.

N-Sink was developed as a web-based tool to visualize and explore landscape N sinks at the HUC-12 scale (HUC = Hydrologic Unit Code; ( Berelson et al. 2004)) and makes extensive use of widely available GIS data. The theoretical underpinnings are outlined in Kellogg et al. (2010) and build on peer-reviewed meta-analyses and reviews ( Seitzinger et al. 2006, Alexander et al. 2007, Mayer et al. 2007) to estimate N removal within landscape N sinks – wetlands, streams and ponds. The approach relies on residence time within landscape features that support N removal as the primary driver of N retention and transformation (e.g., Klocker et al. 2009).

The *nsink* R package was written to simplify the acquisition and management of the spatial data necessary to estimate N removal within each identified landscape sink, estimate cumulative N removal along a specified flowpath, and create a set of static watershed maps ( Hollister et al. 2022). Datasets used by *nsink* include the National Hydrography Dataset Plus (NHDPlus), Soil Survey Geographic Database (SSURGO), the National Land Cover Dataset (NLCD) land cover and the National Land Cover Dataset (NLCD) impervious surface ( Soil Survey Staff 2017, Jin et al. 2019, Moore et al. 2019). The static maps illustrate 1) N Removal Efficiency, a color-coded map of the estimated N removal efficiency of the different landscape sinks; 2) N Transport Index, a heat map with the cumulative N removal along flowpaths originating from sources represented by a grid of points across a watershed. This heat map highlights “leaky” source areas with less downstream N retention versus those with higher downstream retention; and 3) N Delivery Index, combining maps (1) and (2) along with source loading based on NLCD categories and expressed as an index ranging from 0 to 1, resulting in a map showing potential N delivery from different sources, after accounting for the potential N removal as water moves downstream. N-Sink and *nsink* integrate these data to make the application of complex hydrologic and biogeochemical processes accessible to end users and stakeholders in a visually clear and useful format. Here we describe the development of the package, and example workflow, and simple use applications to demonstrate the capabilities of the tools. The code for the analyses presented here is available from https://github.com/USEPA/nsink_manuscript/ and is archived at https://doi.org/10.5281/zenodo.10045141 ( Kellogg et al. 2023). All analyses were conducted with R version 4.2.2 and details on R package versions and operating system used for this analysis are included in a file, sessioninfo.txt at https://github.com/USEPA/nsink_manuscript/blob/main/sessioninfo.txt ( Grolemund and Wickham 2011, Xie 2014, 2015, 2023, Pebesma et al. 2016, Wickham 2016, Pebesma 2018, Müller 2020, Hollister et al. 2022, R Core Team 2022, Bocinsky 2023, Hernangómez 2023, Hijmans 2023a, 2023b, Pebesma and Bivand 2023).

## Methods

### Implementation in R

The package *nsink* implements the approach detailed in Kellogg et al. (2010) to estimate relative nitrogen (N) removal along a flowpath. The *nsink* package follows from an initial version of N-Sink written in ArcGIS using ModelBuilder. The initial version required time-consuming data manipulation by hand due to limitations of earlier NHDPlus datasets as well as the limitations of working in a GIS environment requiring a user license. With the increasingly more versatile GIS packages now available in R Project for Statistical Computing (RRID:SCR_001905), the previously time-consuming spatial data acquisition and management of N-Sink was automated and applied to other HUC-12 catchments, leading to the development of the *nsink* package. Specifically, *nsink* relies on packages *sf* and *terra.* The *nsink* package is available from https://github.com/usepa/nsink and is fully described in ( Hollister et al. 2022).

### Using *nsink*

The *nsink* package provides several functions ( Table 1) to set up and run an N-Sink analysis for a specified 12-digit HUC. By using the package all required data are downloaded, prepared for the analysis, HUC-wide N removal calculated, and flowpaths summarized. Additionally, a convenience function, nsink_build(), is included that will run all of the required functions for a specified HUC. The workflow follows a simple series of steps to create a set of static maps: N Removal Efficiency, N Transport Index, and N Delivery Index.

**Table 1.** Functions in the R package *nsink*, with short descriptions.

Function	Description
nsink_get_huc_id	Takes Hydrologic Unit Code (HUC) Name and returns HUC12 IDs
nsink_get_data	Downloads local copies of datasets for specified HUC
nsink_prep_data	Standardizes projections, clips all datasets to HUC boundary
nsink_calc_removal	Calculates percentage of nitrogen (N) removal within the various landscape N sinks
nsink_generate_flowpath	Generates a flowpath from XY starting point for use in N removal analysis
nsink_summarize_flowpath	Estimates N removal along flowpath and lists removal by type of N sink encountered
nsink_generate_static_maps	Generates static maps for a given HUC
nsink_plot	Creates simple plot with pre-selected palettes from a list of static maps created by nsink_generate_static_maps()
nsink_build	Wrapper function that runs all required functions to build the full dataset and static maps for a specified HUC12

### Download the data

The first step for an N-Sink analysis with the *nsink* package is to download the required datasets. The only required information to download the data is a HUC identifier. The *nsink* package was developed using 12-digit HUC IDs from NHDPlus, but larger HUCs (e.g. 8-digit) may also be used. There are two functions provided for downloading the data.

• nsink_get_huc_id() - search 12-digit HUC IDs using a known location name.
• nsink_get_data() - download and save, in a specified folder, hydrography, soils, and land cover data for the specified 12-digit HUC. These data cover the HUC, but have not been pared down to those data exclusive to the specified HUC. The *nsink* package utilizes widely available data from several U.S. Federal Government sources and as of 2023-09-30 no authentication is required to access these sources. The HUC ID is required and users may specify a path for storing the data as well as indicate whether or not to download the data again if they already exist in the data directory.

### Prepare the data

Once the data are downloaded there are several additional data processing steps that are required to subset the data to the HUC and set all data to a common coordinate reference system (CRS).

These include:

• filter out the HUC boundary
• mask all other data sets to the HUC boundary
• convert all column names to lower case
• create new columns
• harmonize raster extents
• set all data to common CRS

The nsink_prep_data() function will complete all of these processing steps. It requires a HUC ID, a specified CRS, and a path to a data directory. It returns a “list” (a specific data format in R) with all required data for subsequent N-Sink analyses.

### Calculate N removal

The next step in the N-Sink workflow is to calculate potential nitrogen removal within each landscape sink, expressed as percent of incoming N. Details on how the nitrogen removal estimates are calculated are available in Kellogg et al. (2010), and make use of peer-reviewed literature. Since the publication of that article, changes have been implemented to make better use of the most recently available geospatial data. Those changes are described below. The nsink_calc_removal() function takes the prepared data as an input and returns a “list” with three items:

• raster_method: Contains a raster-based approach to calculating removal. Used for the static maps showing potential N removal within landscape sinks.
• land_removal: Represents land-based nitrogen removal within vegetated hydric soils with impervious surface removed.
• network_removal: Contains removal along the NHD Plus flow network. Removal is calculated separately for streams and waterbodies (i.e., lakes and reservoirs).

### Generate and summarize flowpaths

A useful part of the N-Sink approach is not just the calculation of potential N removal for individual components of the landscape, it is the ability to summarize cumulative removal along the length of a specified flowpath. Starting from any specified location the flowpath on land is generated from a flow direction grid. Once that flowpath intersects the surface water network, flow is determined by flow along the NHD Plus surface water network. With a flowpath generated, the cumulative N removal along that flowpath can be calculated with the nsink_summarize_flowpath() function, taking the flowpath and removal as input. A data frame is returned with each segment identified by type of sink, if present (i.e., “Hydric”, “Stream”, “Lake/Pond”, or “No Removal”), the percent removal associated with that segment, and cumulative removal. Total cumulative removal is 100 - the minimum of the n_out column. That is, n_out keeps track of the percent of N leaving each subsequent downstream segment ( Table 2). Note that the most downstream pond and stream segments show essentially no N removal due to a short retention time.

**Table 2.** Summary of N removal along specified flowpath A (see Figure 4), as generated by the R package *nsink.*

segment_type	length_meters	percent_removal	n_in	n_out
Hydric	336	44.000	100.0	56.0
Stream	1518	0.120	56.0	55.9
Stream	462	0.007	55.9	55.9
Stream	1202	0.016	55.9	55.9
Stream	653	0.007	55.9	55.9
Stream	77	0.002	55.9	55.9
Stream	2164	0.041	55.9	55.9
Stream	2246	0.024	55.9	55.9
Lake/Pond	130	0.000	55.9	55.9
Stream	1088	0.010	55.9	55.9
Lake/Pond	462	0.000	55.9	55.9
Stream	59	0.000	55.9	55.9
Lake/Pond	5894	0.000	55.9	55.9

### Static maps

Individual flowpaths are useful for specific areas of interest, but it is also useful to look at removal patterns across the landscape. The nsink_generate_static_maps() function produces a set of HUC-wide raster maps. Required inputs are the prepped data, removal raster, and sampling density, for which a default is provided. The function returns four separate rasters.

• removal_effic: HUC-wide estimate of potential nitrogen removal from different landscape sinks as a percentage.
• loading_idx: A normalized index of nitrogen loads by land cover class derived from published sources, ranging from 0 to 1.
• transport_idx: N transport for a sample of all possible flowpaths in a given HUC. This is an expensive computational task, so *nsink* generates a removal hotspot map based on a sample of flowpaths and the final hotspot map is interpolated from these samples and referred to as the nitrogen transport index. The samp_density argument controls the number of sample flowpaths generated and is roughly the distance between points in a uniform grid. Each of these starting points is assigned the value of N delivery (%) based on cumulative N removal along the flowpath from that starting point. The points are then interpolated with an inverse distance weighted interpolation to a roughly 30 meter resolution. A sampling density of 300 was used to create the static maps in the web application.
• delivery_idx: The delivery index is the combination of the loading index and the transport index. It highlights areas of the landscape that are delivering the most nitrogen to the outflow of the watershed based on NLCD land cover data and transport efficiency.

**Figure 1.**
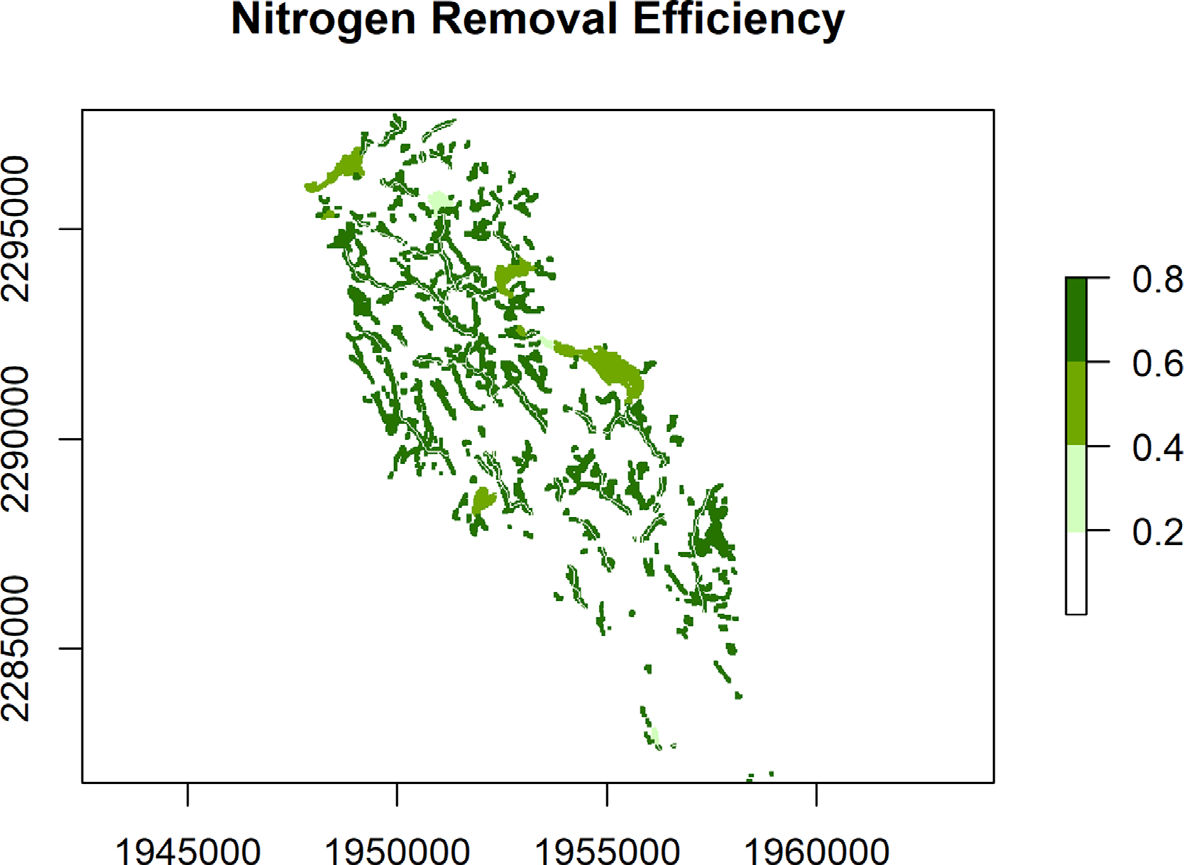
Nitrogen Removal Efficiency (range 0 to 1) for the Niantic River HUC-12, showing the estimated fraction of N removed within three types of landscape N sinks - wetlands, streams, lakes/ponds/reservoirs.

**Figure 2.**
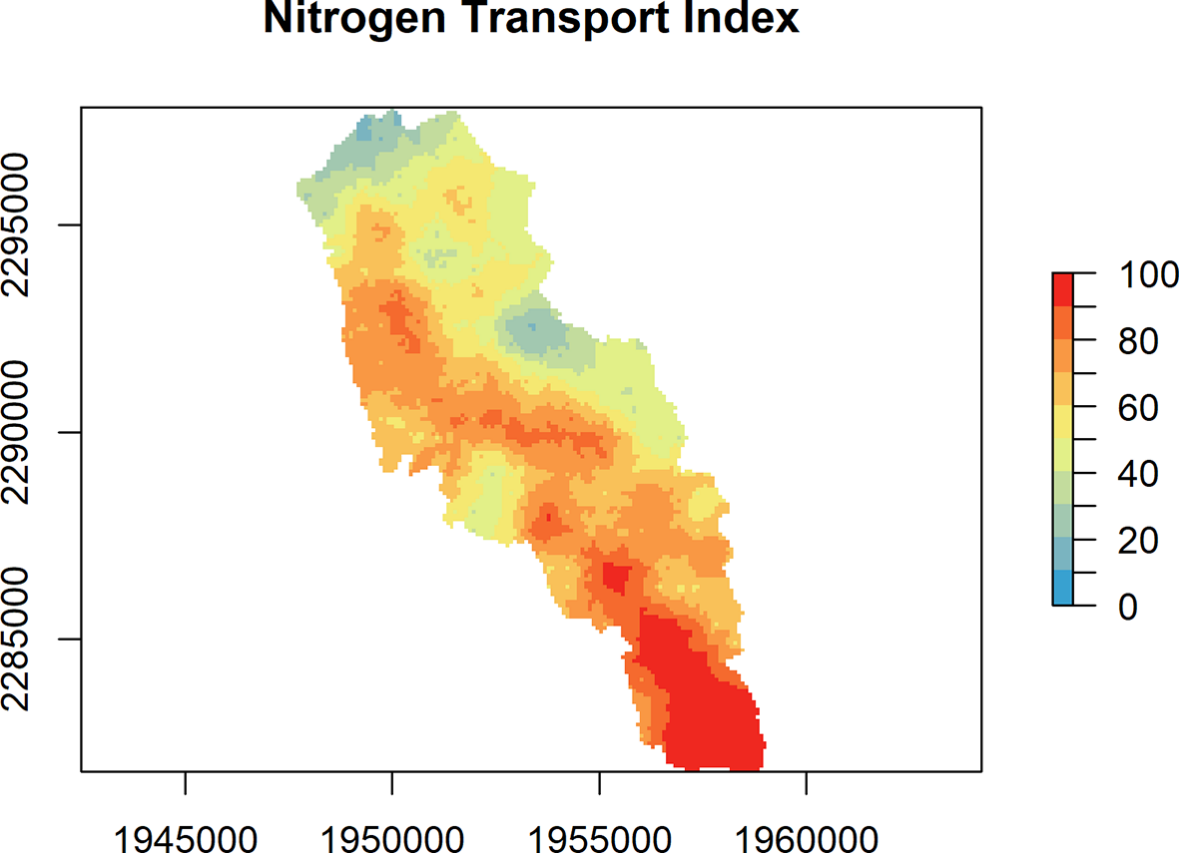
Nitrogen Transport Index (range 0 to 100%) for the Niantic River HUC-12, showing the estimated percentage of N originating at a given location within a watershed that is expected to reach downstream receiving waters, such as a coastal embayment.

**Figure 3.**
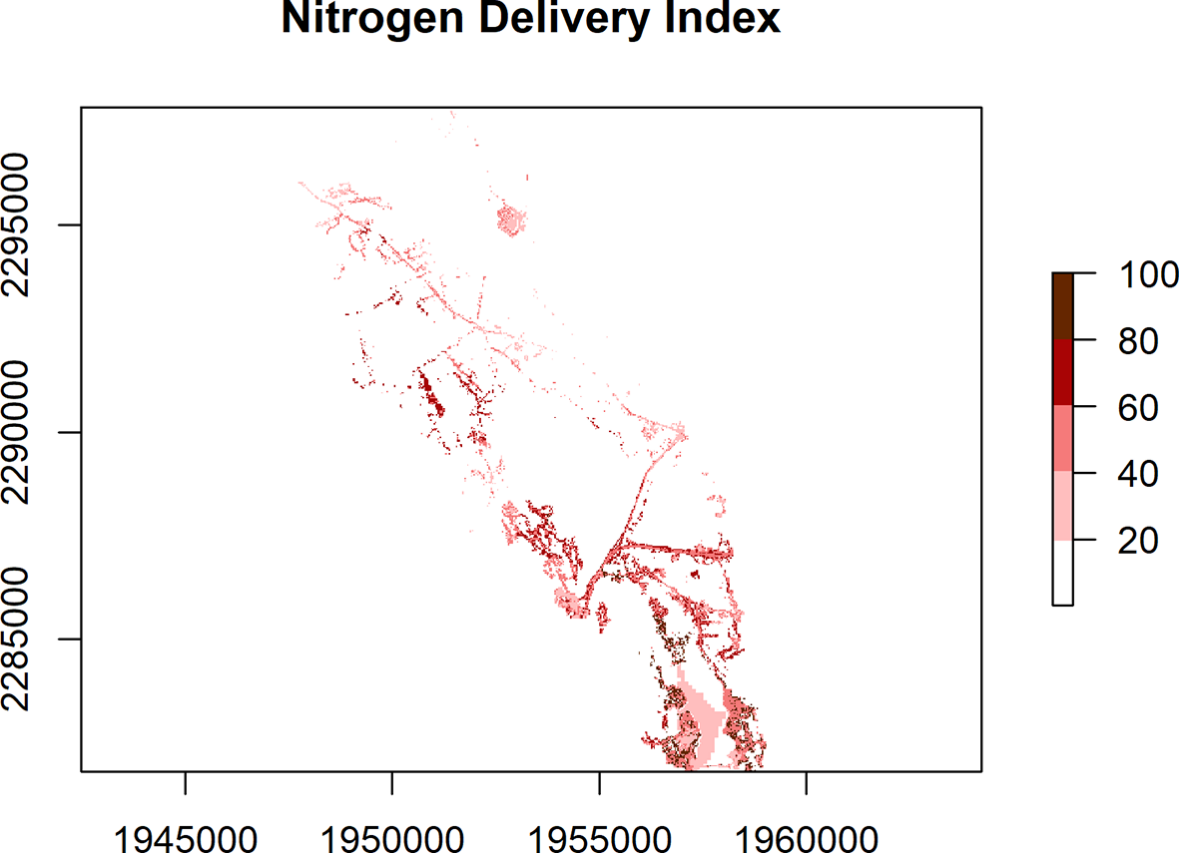
Nitrogen Delivery Index (range 0 to 100%) for the Niantic River HUC-12, showing a relative measure of the estimated N being transported from a location within a mapped watershed to receiving waters. N loading estimates are based on land cover classes, making use of peer-reviewed published data, and normalized, with a range of 0 to 1. These N loading factors are then multiplied by the N Transport Efficiency (range 0 to 100) from a given location within a watershed, to arrive at the N Delivery Index.

These static maps can be quickly plotted with the convenience function nsink_plot() using the map argument to identify map type. For example, nsink_plot(niantic_static_maps, map = "removal") will create a plot of Nitrogen Removal Efficiency ( Figure 1). The other map argument options are “transport” and “delivery” ( Figures 2 and 3). These plots are designed for exploration and not necessarily for high quality maps. For higher quality maps, the source data can be plotted in software of a user’s choice (e.g. QGIS, ggplot2).

### Convenience function to build it all

The workflow described above includes all the basic functionality. Some users may wish to use *nsink* to calculate the base layers for an N-Sink analysis and then build an application outside of R. A convenience function that downloads all data, prepare the data, calculates removal, and generates static maps has been included to facilitate this type of analysis. The nsink_build() function requires a HUC ID, coordinate reference system, and sampling density. An output folder is also needed but has a default location. There is an optional argument for forcing a new download. All prepped data shape files and .tif files are written to the output folder for use in other applications.

N-Sink maps are now available for HUC-12 catchments in coastal CT, RI and southern MA as a web application interactive tool. This app can be used to examine and better understand N movement through these HUC-12 catchments.

### Changes to underlying methodology

Several improvements have been made to the underlying N-Sink methodology used in Kellogg et al. (2010) that have allowed *nsink* to make use of more recently available spatial data. In estimating N removal in streams, we originally estimated stream depth and velocity based on other available geospatial data to arrive at a travel time along a given stream reach. With the updated and expanded NHD Plus V2 we use the estimates provided in that dataset, making use of USGS expertise that went into these estimates.

In estimating N removal occurring in the terrestrial portion of the flowpath, we originally focused on riparian wetlands, using SSURGO mapped hydric soils to identify this landscape sink. With the latest version of SSURGO we include all hydric soils in the catchment, except those identified as “subaqueous”. Each soil mapping unit (SMU) that has a positive hydric status also has an estimate of %hydric for that SMU. We then estimate N removal within that SMU as 80% of the %hydric. Each raster has one SMU associated with it and raster cell size is 30m X 30m. A 30-m wide wetland is estimated by Mayer et al. (2007) as having 80% N removal. In the current version, N removal is cumulative as a flowpath intercepts hydric soils in any cell before encountering surface water.

### Off-network hydrology

In the NHD Plus dataset there are situations where a water body or stream segment is “off-network”, i.e., it is not linked to the larger surface flow network. These may be features such as groundwater-fed kettle ponds, or canals or ditches where connecting hydrology may not be captured in the NHD Plus dataset. These off-network features tend to have less NHD data (e.g., time of travel) associated with them, making direct N removal estimates impossible. In these cases, we must estimate removal based on other in-network surface water features. For the vast majority of HUC-12 catchments off-network features are rare and spatially small compared to the overall system. We examined seven HUC-12s within southern New England, with a range of land cover and soils, for the extent of off-network hydrology. None had off network stream or canal segments, while six of the seven had off-network waterbodies. These off-network waterbodies represented from 2 to 14% (mean of 7%) of total lake area. We estimated removal from off-network stream segments to be equal to the median removal of all first order streams in the same catchment. In this case we are assuming these first order segments behave similarly to other first order stream reaches. A subset of these off-network stream segments is identified by the NHD dataset as ditches and canals. These are expected to provide little N removal due to their designed intent to move water efficiently, reducing residence time ( Buchanan et al. 2013), and therefore N-Sink assigns removal as the lower quartile of removal from the highest order streams in the same catchment. Finally, off-network lakes and ponds are assigned N removal at the 3rd quartile of removal from all lakes and ponds in the same catchment due to the level of uncertainty regarding residence time in these groundwater-fed lakes and ponds ( Hampton et al. 2019).

### Caveats

N-Sink was designed for catchments where surface and groundwater flow paths are not highly manipulated. In settings where substantial storm water conveyance networks or extensive subsurface agricultural drainage are present, flow can bypass the removal processes within wetland ecosystems ( Gold et al. 2001). In these situations, first-order streams may be channelized, leading to higher velocities than in undisturbed channels and the N-Sink estimates of removal may be overstated. N-Sink does not account for nitrogen removal from high permeability (e.g., sand and gravel) unconfined aquifers along the coastal margin. Here, nitrogen-enriched groundwater may enter estuaries directly as submarine discharge rather than via baseflow to stream networks ( Nowicki and Gold 2008), requiring site specific assessments to evaluate N removal in salt marshes or as upwelling into the seabed ( Addy et al. 2005, Robinson et al. 2018). These situations have been estimated to account for approximately 6% of freshwater inputs to estuaries globally and in areas of New England ( Burnett et al. 2003, Nowicki and Gold 2008).

The variation in loading associated with different agricultural practices, density of unsewered development and inputs from wastewater treatment plants are not encompassed in the default loading index. Thus the numeric outputs of the tool focus on metrics suitable for planning purposes, such as percent removal of N from source to receiving water and relative intensity of loading from different locations. It is not intended to replace high-resolution models that rely on long term weather records and incorporate detailed spatial data on N loading, within-field transport or variable source hydrology (e.g., Easton (2008)). Instead, N-Sink combines widely available datasets for waterway networks, soils and land cover with nitrogen dynamics developed for these datasets to highlight major sources and sinks of nitrogen within a watershed context.

### Operation

The R package *nsink* requires R version >= 3.5.0. The *nsink* package is available from https://github.com/usepa/nsink and is fully described in ( Hollister et al. 2022).

## Use Cases

### Use Case 1: Comparison of N delivery from two adjacent locations

Users can use the following code to interactively identify a starting point for a flowpath of interest.
*
# Plot watershed with base R*
plot (st_geometry(niantic_data$huc))

*# Select location on map for starting point using cursor and click*
pt <- unlist (locator(n = 1))

*# Convert to sf POINT*
start_loc <- st_sf(st_sfc(st_point(pt), crs = aea))

*# Generate the flowpath*
niantic_fp <- nsink_generate_flowpath(start_loc, niantic_data)

*# Plot flowpath*
plot (st_geometry(niantic_fp$flowpath_network), add = TRUE)

*# Summarize N removal along the flowpath*
niantic_removal <- nsink_calc_removal(niantic_data)

*# table of removal within flowpath segments*
niantic_fp_removal <- nsink_summarize_flowpath(niantic_fp, niantic_removal)


**Figure 4.**
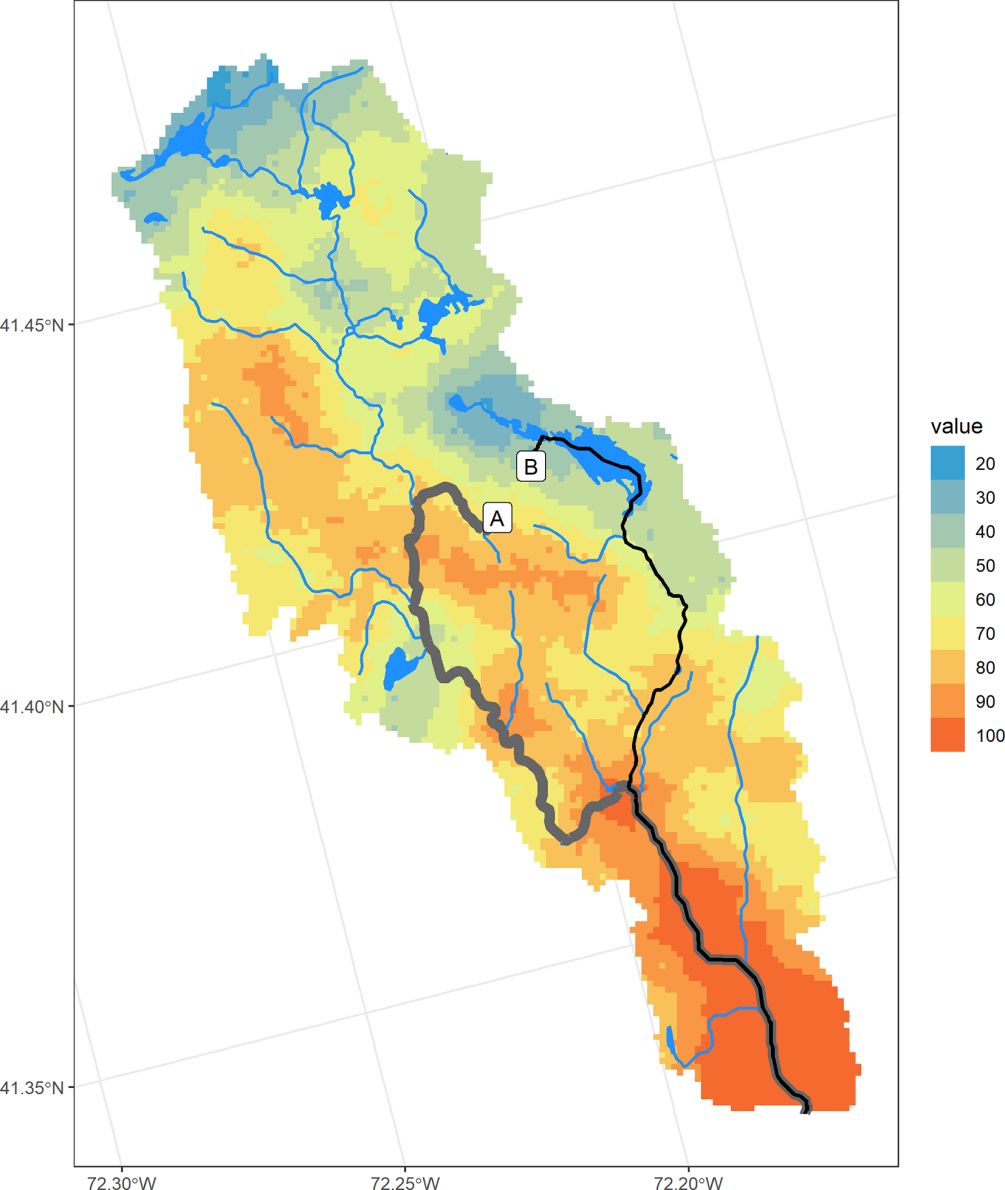
Flowpaths originating from two nearby locations within the Niantic River HUC-12 with differing N removal estimates. Hydrofeatures are pictured on the Nitrogen Transport Index map.

Using this approach, we can compare the N delivery from two locations that are separated by approximately 900 meters. This example illustrates the importance of location with respect to landscape N sinks. Approximately 56% of the N that originates at point A is transported to the estuary vs. approximately 25% of the N that originates at point B ( Figure 4). The difference is due to the different N sinks encountered along the way to the estuary. Most of the removal that occurs along flowpath A is due to flow through hydric (wetland) soils, with little removal occurring during flow along the downgradient stream network ( Table 2). Flowpath B encounters a large pond along the way, significantly increasing residence time and N transformation ( Table 3). These two currently undeveloped locations demonstrate the importance of understanding landscape position and its role in the movement of N from source to outlet. If development were proposed in the western portion of this area (flowpath A), source controls could be advised, while the less “leaky” eastern region (flowpath B) underlines the importance of wetlands and slow-moving water ways, such as ponds, that protect downstream waters by slowing flow and increasing opportunities for N transformation.

**Table 3.** Summary of N removal along specified flowpath B (see Figure 4), as generated by the R package *nsink.*

segment_type	length_meters	percent_removal	n_in	n_out
Hydric	287	4.390	100.0	95.6
No Removal	276	0.000	95.6	95.6
Lake/Pond	84	41.400	95.6	56.0
Stream	61	0.005	56.0	56.0
Lake/Pond	2170	56.300	56.0	24.5
Stream	316	0.022	24.5	24.5
Stream	2480	0.076	24.5	24.4
Lake/Pond	167	0.000	24.4	24.4
Stream	816	0.022	24.4	24.4
Stream	1056	0.022	24.4	24.4
Lake/Pond	338	0.000	24.4	24.4
Stream	59	0.000	24.4	24.4
Lake/Pond	5894	0.000	24.4	24.4

### Use Case 2: Source controls and sink protection or restoration

The maps produced by N-Sink can help prioritize source controls, sink protection and/or restoration. For example, many wetlands are considered “protected” by federal, state or local laws and regulations; but as occurs with many types of protected areas ( Neal et al. 2018) they are frequently altered through variances granted by local commissions or committees charged with zoning decisions. By better understanding the important role that N sinks play in the protection of estuaries, local agencies have a better chance of achieving protection of downstream receiving waters. Variances granted on a case-by-case basis can quickly undermine local water quality goals. Land conservancies and other NGOs interested in identifying parcels of high value for protecting downstream waters from N loading can also use these maps to prioritize land acquisition or wetland restoration projects.

**Figure 5.**
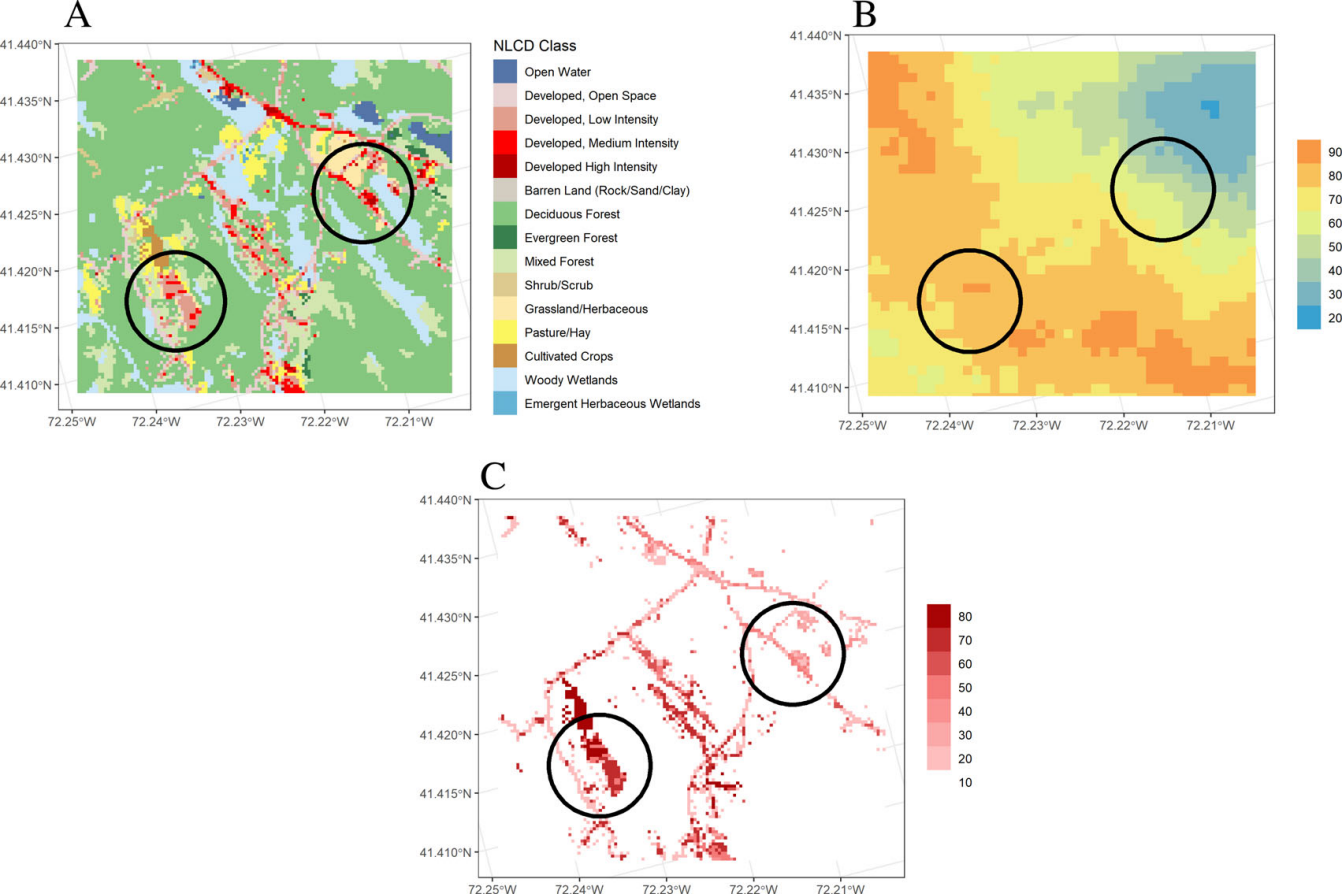
Comparison of two similarly developed areas with differing N delivery indices in the Niantic River HUC-12. A. Land cover, summarized in Table 4. B. Nitrogen Transport Index. Hotter colors denote higher values. C. Nitrogen Delivery Index. Darker colors denote higher N delivery based on normalized loading from different land covers, multiplied by the N Transport Index.

Similarly, sources of N that are contributing relatively more N due to their location within the catchment can be highlighted using the *nsink* static maps. The N Delivery Index map combines N sources with N transport to highlight developed areas that are located on “leakier” parts of a catchment. Figure 5A displays a high resolution image of land cover in the same watershed as Example 1, with two developed areas circled. Figure 5B displays the Nitrogen Transport Index map for the same area, showing the western area to be “leakier” than the eastern area.

**Table 4.** Summary of land cover in highlighted areas of interest ( Figure 5).

Land Cover	West (%)	East (%)
Developed	23	20
Agriculture	7	0
Forest/Vegetated	64	61
Wetlands	6	18
Other	0	1

An analysis of N delivery from these two similarly developed areas (average N loading index of developed areas: West = 0.57; East = 0.56), show the western area may contribute more N loading to the estuary than the eastern area, with the average delivery index value of the western area (41) almost 80% higher than the eastern area (23; Figure 5C), suggesting a greater need for N source controls within the western development.

Other examples using N-Sink to examine the N loading implications of past land use decisions are also available as bonus maps on the N-Sink website.

## Discussion

N-Sink as implemented in the *nsink* R package allows users to easily download all necessary spatial data (hydrography, land cover and soils), create static maps (N Removal Efficiency, N Transport Efficiency and N Delivery Index), and interactively investigate N removal along flowpaths within any specified catchment. Currently, the default catchment definition uses HUC-12 designations, though it is also possible to use larger catchments. Larger catchments would require longer download and processing times. For investigators who do not work in R, we have created a web application that covers HUC-12 catchments in coastal CT, RI and southern MA using an interactive tool.

N-Sink is a decision support tool and was created to be a useful, easy way for local land use managers to explore the relationship between land use and nitrogen pollution in their waters. Currently N-Sink is being used as an integral component in a project based in Long Island Sound, funded by the Long Island Sound Study Research Fund. The project is focused on targeting outreach on fertilizer use within the 43,560 km ^2^ catchment. Local decision-makers that have prioritized nitrogen mitigation in their long-term planning efforts can use this tool to help them better understand the potential impact of proposed development projects and zoning variances. Similarly, land trusts and other NGOs interested in N mitigation can use this tool to identify high priority areas for acquisition or restoration.

## Software availability

Software available from: https://github.com/usepa/nsink

Source code available from: https://github.com/usepa/nsink

Archived source code at time of publication https://doi.org/10.5281/zenodo.10045141 ( Kellogg et al. 2023)

License: *MIT*

## Data Availability

Datasets used by nsink include the National Hydrography Dataset Plus (NHDPlus; Moore et al. 2019), Soil Survey Geographic Database (SSURGO; Soil Survey Staff 2017), the National Land Cover Dataset (NLCD) land cover and the National Land Cover Dataset (NLCD) impervious surface ( Jin et al. 2019). All are available via the FedData package in R ( Bocinsky 2023).
