# Supplementary material for: Using the R Package, nsink, to assess landscape N removal in coastal catchments
Source: F1000Res. 2024 Nov 6;13:595. Originally published 2024 Jun 7. [Version 2] doi: 10.12688/f1000research.144100.2 (PMC11582390; doi:10.12688/f1000research.144100.2)
Supplement: Peer review history

# Reviewer report: Reviewer response for version 2

**DOI:** 10.5256/f1000research.172299.r338325

**Published:** 2024-11-21

**Authors:** Lora Harris

**Affiliations:** 1 University of Maryland Center for Environmental Science,, Solomons,, Maryland,, USA

Thank you for responding to my review.

Are the conclusions about the tool and its performance adequately supported by the findings presented in the article?

Yes

Is the rationale for developing the new software tool clearly explained?

Yes

Is the description of the software tool technically sound?

Yes

Are sufficient details of the code, methods and analysis (if applicable) provided to allow replication of the software development and its use by others?

Yes

Is sufficient information provided to allow interpretation of the expected output datasets and any results generated using the tool?

Yes

Reviewer Expertise:

coastal ecology

I confirm that I have read this submission and believe that I have an appropriate level of expertise to confirm that it is of an acceptable scientific standard.

# Reviewer report: Reviewer response for version 2

**DOI:** 10.5256/f1000research.172299.r338326

**Published:** 2024-11-09

**Authors:** Jianning Ren

**Affiliations:** 1 Civil and Environment Engineer, National University of Singapore (Ringgold ID: 37580), Singapore, Singapore, Singapore

All my concerns have been addressed well, I agree the manuscript to be accepted for indexing.

Are the conclusions about the tool and its performance adequately supported by the findings presented in the article?

Partly

Is the rationale for developing the new software tool clearly explained?

Yes

Is the description of the software tool technically sound?

Yes

Are sufficient details of the code, methods and analysis (if applicable) provided to allow replication of the software development and its use by others?

Partly

Is sufficient information provided to allow interpretation of the expected output datasets and any results generated using the tool?

Partly

Reviewer Expertise:

Ecohydrology

I confirm that I have read this submission and believe that I have an appropriate level of expertise to confirm that it is of an acceptable scientific standard.

# Reviewer report: Reviewer response for version 1

**DOI:** 10.5256/f1000research.157845.r296724

**Published:** 2024-08-23

**Authors:** Lora Harris

**Affiliations:** 1 University of Maryland Center for Environmental Science,, Solomons,, Maryland,, USA

Having the nsink tool available in R is a wonderful advancement for this approach to evaluating nitrogen sources in coastal watersheds. I have only minor suggestions to improve description of this tool in this venue.

 There is a bit of confusion between the title of the article and then the in depth description of nsink itself alongside the published Hollister et al. reference. I am familiar with the Kellogg 2010 work upon which this is based, and in looking at the Hollister reference and github code, I think what I am discerning is that the Hollister  et al. (2022) reference is the publication of the actual code, and the hope for this article is that it will showcase how to implement and use the software tool.  I believe that there could be some slight improvement to wording to better clarify that. As it stands, the introduction largely introduces nsink rather than the analyses. I believe an additional paragraph or tightening of the third paragraph to clearly transition into setting up the reader to understand the analyses will help to better connect the title, nsink/Hollister et al. (2022) resource, and the work focused on here.

 Likewise, in the methods if there is a way to specify the regional setting for the case study examples or somehow write in a way that clarifies that you are showcasing a case study for the Niantic River, then it will connect better to both the title and resulting figures. Something like an opening paragraph that states clearly that you are presenting a case study for the Niantic River as a way to demonstrate the utility of nsink and that the methods are generalizable to other systems? The Use Cases section doesn't really introduce us to the study location.

 I do really love the caveats section. I think that is so important and useful and I applaud the authors for clearly stating the limitations of the model - all models have limitations and those are best understood by the model creators.

Are the conclusions about the tool and its performance adequately supported by the findings presented in the article?

Yes

Is the rationale for developing the new software tool clearly explained?

Yes

Is the description of the software tool technically sound?

Yes

Are sufficient details of the code, methods and analysis (if applicable) provided to allow replication of the software development and its use by others?

Yes

Is sufficient information provided to allow interpretation of the expected output datasets and any results generated using the tool?

Yes

Reviewer Expertise:

coastal ecology

I confirm that I have read this submission and believe that I have an appropriate level of expertise to confirm that it is of an acceptable scientific standard, however I have significant reservations, as outlined above.

# Response

**Published:** 2024-10-29

**Authors:** Dorothy Kellogg

**Affiliations:** University of Rhode Island, Kingston, Rhode Island, USA

Thank you very much for your thoughtful feedback. In response to your comments:

 1. We have changed the title to better reflect the main focus of the article.

 2. We have added a brief introduction to the Use Cases describing their location. We have also added to the narrative so that it is clear that this tool can be used in any other appropriate HUC because the data are widely available.

 Many thanks

# Reviewer report: Reviewer response for version 1

**DOI:** 10.5256/f1000research.157845.r296733

**Published:** 2024-08-10

**Authors:** Jianning Ren

**Affiliations:** 1 Civil and Environment Engineer, National University of Singapore (Ringgold ID: 37580), Singapore, Singapore, Singapore

In this paper, the authors developed a decision-supporting tool, N-Sink, based on the R package nsink. They described the motivation behind developing nsink, and presented two use case examples of implementing nsink R package. The manuscript is well-written, and the tool can be helpful to local decision-makers. However, I have two main concerns: 1. Since this is a research paper, the author should include some paragraphs discussing how this tool can help with research and the scientific contribution of this research besides helping with decision-making. The web interface is an excellent application; the authors should put some effort into emphasizing this part. So I suggest accept with minor revisions.

 Below are some detailed comments:

1. Should put line numbers in the manuscript
2. The introduction should introduce the N-sink web-based tool earlier with a link and explain the differences between N-sink and nsink
3. The third paragraph, how will the authors deal with the R package issues, such as “raster” package is deprecated and replaced by “terra”. Or how to deal with the version updating issues of R packages. 
4. I have tested the N tracker functionality in the web application, there is an error, I suggest to fix this:" please install the ArcGIS R intergration package R version 4.4.1" failed to execute N tracker V13

Are the conclusions about the tool and its performance adequately supported by the findings presented in the article?

Partly

Is the rationale for developing the new software tool clearly explained?

Yes

Is the description of the software tool technically sound?

Yes

Are sufficient details of the code, methods and analysis (if applicable) provided to allow replication of the software development and its use by others?

Partly

Is sufficient information provided to allow interpretation of the expected output datasets and any results generated using the tool?

Partly

Reviewer Expertise:

Ecohydrology

I confirm that I have read this submission and believe that I have an appropriate level of expertise to confirm that it is of an acceptable scientific standard, however I have significant reservations, as outlined above.

# Response

**Published:** 2024-10-29

**Authors:** Dorothy Kellogg

**Affiliations:** University of Rhode Island, Kingston, Rhode Island, USA

Thank you for your very helpful feedback. In response to your two main concerns we have a) added to the narrative to describe ways that the tool can be helpful in resource economics research and provided examples of similar investigations, and b) amended the introduction and overview to better emphasize the web application. Specifically responding to your numbered comments:

 1. Alas, we do not have control over the line numbering.

 2. We introduce the web application earlier, along with a link, and made changes to the narrative to clarify the differences between N-sink method and the associated R package, *nsink*.

 3. We will be updating the package as changes are made in R. This is an ongoing task with any R package.

 4. Thank you for identifying this problem. We have identified the source of the problem and have addressed it. You may confirm that things are working here.

 Many thanks
